# Urinary Naphthol as a Biomarker of Exposure: Results from an Oral Exposure to Carbaryl and Workers Occupationally Exposed to Naphthalene

**DOI:** 10.3390/toxics5010003

**Published:** 2017-01-06

**Authors:** Craig Sams

**Affiliations:** Health and Safety Executive, Harpur Hill, Buxton SK17 9JN, UK; craig.sams@hsl.gsi.gov.uk; Tel.: +44-129-821-8070

**Keywords:** carbaryl, exposure assessment, human biomonitoring, naphthalene, naphthol

## Abstract

Urinary naphthol is an established human biomarker used for assessing both occupational and environmental exposure. However, 1-naphthol is a metabolite of the insecticide carbaryl while both the 1- and 2-isomers are metabolites of naphthalene. Thus, urinary 1-naphthol levels will reflect combined exposure to both substances, particularly at environmental levels. The interpretation of biomarkers is aided by knowledge of levels following well-characterised exposure scenarios. This study reports urinary 1-naphthol levels in five volunteers administered an oral dose of carbaryl at the acceptable daily intake (ADI, 0.008 mg/kg). The elimination half-life was 3.6 h and the mean 1-naphthol level in 24 h total urine collections, normalised for a 70 kg individual, was 37.4 µmol/mol creatinine (range 21.3–84.3). Peak levels in spot-urine samples were around 200 µmol/mol creatinine. For comparison, 327 post-shift urine samples obtained from 90 individual workers exposed occupationally to naphthalene had 1-naphthol levels from below the limit of detection (<LoD) to 1027 µmol/mol creatinine (median = 4.2, mean = 27.2). The 2-naphthol levels ranged from <LoD to 153 µmol/mol creatinine (median = 4.0, mean = 8.1). Background ranges have been reported for urine naphthols in several populations, with upper limits between 10 and 20 µmol/mol creatinine. The data reported here suggest that environmental exposure to carbaryl and naphthalene in these populations is well controlled.

## 1. Introduction

Carbaryl (1-naphthyl-methylcarbamate; CAS No. 63-25-2) is a carbamate insecticide used on a variety of crops [1,2]. Workers involved in the formulation or application of products containing carbaryl may be exposed either dermally or via inhalation. The general public may be exposed via spray drift in areas immediately surrounding treated crops, while more widespread dietary exposure may occur from eating contaminated foods. Alongside many other pesticides, carbaryl continues to be linked with a range of potential adverse health effects including neurological issues [3,4] and cancer [5,6]. Consequently, a precautionary approach is generally taken to ensure that human exposures are well controlled. Acceptable daily intakes (ADIs) are used to assess risk to food consumers. Carbaryl has an ADI of 0.008 mg per kg of body weight per day [7]. 

In humans, carbaryl is quickly metabolised to 1-naphthol which undergoes conjugation and is subsequently excreted in urine [8]. Urinary 1-naphthol has therefore been employed as an exposure biomarker [9,10,11]. Biological monitoring, or human biomonitoring, is a useful means of assessing an individual’s total exposure via all routes and has been widely used for monitoring worker exposure and also in wider population studies [12]. The ability to quantify substances, or their metabolites, in non-invasive samples such as urine makes biological monitoring particularly applicable for the relatively simple collection of large numbers of samples. However, there is a need for an appropriate reference range to help with the interpretation of the data. 

However, 1-naphthol is not a specific biomarker for exposure to carbaryl. Conjugates of both 1- and 2-naphthol isomers are major metabolites of naphthalene excreted in urine. Environmental exposure to naphthalene can arise from the combustion of hydrocarbons or tobacco smoke [13,14]. Consequently, background population levels of 1-naphthol will generally reflect environmental and dietary exposure to a combination of both carbaryl and naphthalene. It has been suggested that the ratio of the two naphthol isomers might indicate the relative contribution of the two potential sources [15].

This study reports urinary metabolite levels found in volunteers who received a single oral dose of carbaryl at the ADI. These data will be discussed in relation to naphthol levels measured in the urine of naphthalene-exposed workers as well as background levels in the UK population [16], resulting from environmental exposure to both carbaryl and naphthalene.

## 2. Materials and Methods 

### 2.1. Chemicals

Neat carbaryl and individual naphthol isomers were obtained from QMX Laboratories (Thaxted, UK). All solvents and reagents used were of analytical grade. 

### 2.2. Volunteer Study

The investigations detailed herein were carried out in accordance with the rules of the Declaration of Helsinki (1975). The study protocol was approved by the Health and Safety Executive HSE Research Ethics Committee (project identification code was ETHCOM/REG/06/03) in June 2003. All participants gave informed written consent. A single oral dose of carbaryl at the ADI (0.008 mg per kg of body weight per day) was administered to five volunteers dissolved in ethanol and diluted with a soft drink. Participant details including gender, age, height, weight and body mass index (BMI) are shown in Table 1. Total urine collections were made into a series of individual bottles corresponding to 0–2, 2–4, 4–6, 6–8, 8–12, 12–20, 20–24, 24–28, 28–32, 32–36, 36–44 and 44–48 h post-exposure. The volume of each sample was recorded.An aliquot of urine was retained for analysis. Results were creatinine adjusted to account for variations in hydration.

### 2.3. Sample Preparation

Duplicate 2 mL aliquots of urine were mixed with 100 µL glucuronidase solution (250 µL glucuronidase from *Helix pomatia* (Sigma-Aldrich, Gillingham, UK) in 50 mL 0.1 M acetate buffer (pH 5) and 900 µL acetate buffer and incubated overnight at 37 °C to release free naphthols from conjugates. Calibration standards (0–200 nmol/L) were prepared in urine and analysed with each run.Quality control samples were prepared from a pool of urine obtained from naphthalene-exposed workers and characterised for acceptance criteria and analysed in duplicate after every five duplicate samples. Samples were extracted using solid phase extraction (C18, 1 mL, 100 mg) using the following conditions: condition with methanol (2 mL) and water (2 mL), load sample, wash with water (1 mL) and 30% acetonitrile (1 mL) then elute with 200 µL methanol. This extract was subsequently directly injected (100 µL) for analysis.

### 2.4. Sample Analysis

Samples were analysed by high performance liquid chromatography (HPLC) employing fluorescence detection (excitation 227 nm, emission 430 nm) (Agilent 1100 series LC). A Pursuit XRs C18 column (150 × 4.6 mm, Agilent) was used with an isocratic 65% methanol: 35% water mobile phase at a flowrate of 1 mL/min.

The assay was linear up to at least 200 nmol/L (as judged by a least squares regression coefficient of >0.99). Inter-assay variation of 17% for 1-naphthol and 20% for 2-naphthol was obtained from repeat analysis of quality control samples. The detection limit was 10 nmol/L for both isomers.

### 2.5. Creatinine Analysis

Creatinine was measured in all urine samples using an automated alkaline picrate method using a Cobas Mira (ABX, Montpellier, France) [17,18]. The coefficient of variation for within-day analysis was 1.5% and for between-day analysis was 3% at 6 mM.

The volunteer study data were adjusted for creatinine levels to account for dilution and to better reflect the spot samples typically available for occupational or environmental exposure monitoring studies. 

### 2.6. Urine Naphthol Isomers in Exposed Workers

Post-shift spot urine samples (327) were obtained from 90 workers (eight females, 22 smokers) occupationally exposed to naphthalene in the UK, across industries such as aluminium smelting (*n* = 100), coal tar, including timber treatment (*n* = 59) and chimney sweeps (*n* = 95). Samples were prepared as described above and levels of 1- and 2-naphthol were quantified.

## 3. Results

**Figure 1:**
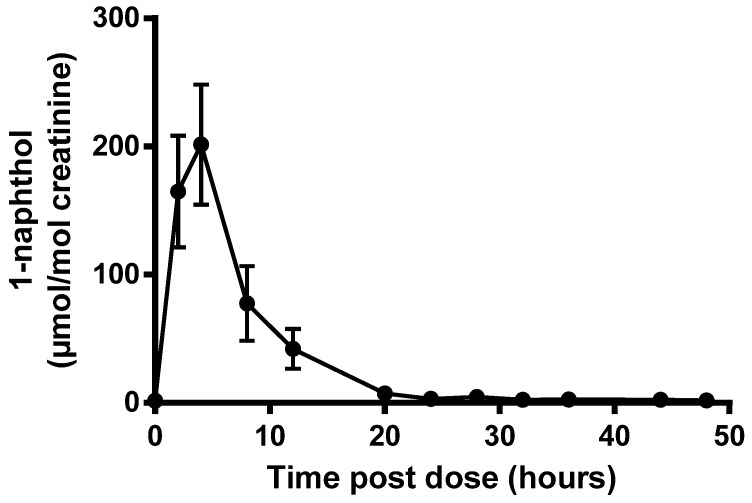
Urinary excretion of 1-naphthol after an oral dose of carbaryl at the ADI. Data were creatinine corrected and individual data points represent the mean ± SD for five volunteers.

**Figure 2:**
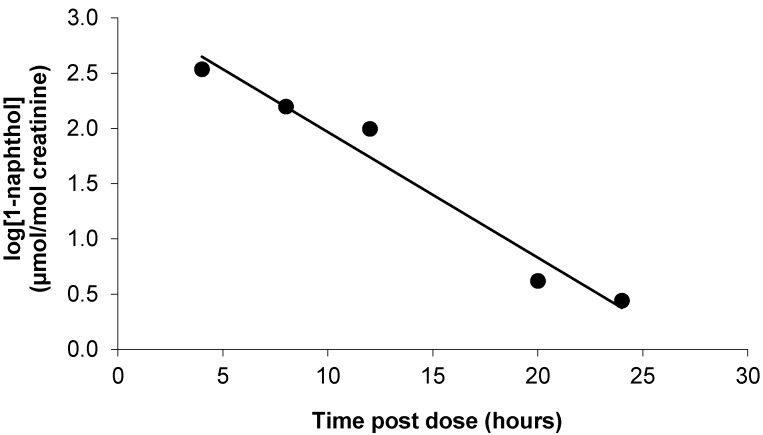
Example of 1-naphthol excretion used for determination of half-life for volunteer A (R^2^ = 0.965). Calculated half-life was 2.6 h.

Following oral administration of carbaryl at the ADI, urinary elimination of 1-naphthol was rapid. Most of the recovered dose was excreted within 24 h of dosing. Figure 1 shows the time course of urinary excretion (mean ± SD) in five volunteers. The peak urinary concentration of 1-naphthol was found between 2 and 6 h post-dose. The mean excretion half-life quantified between 2–4 h and 20–24 h post-dose was 3.6 h (range 2.5–4.8) and an example time course is shown for a single volunteer in Figure 2.

**Figure 3:**
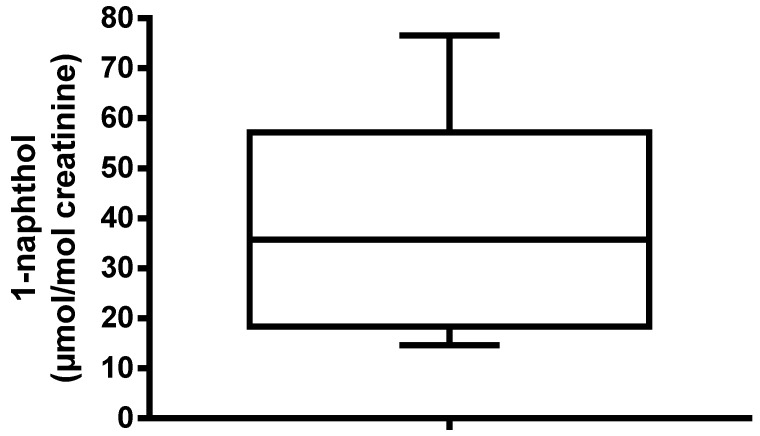
The 1-naphthol levels quantified in 24 h total urine collections from five volunteers given a single oral dose of carbaryl at the ADI. All values are normalised for a 70 kg individual.

An average of 22% (range 11%–43%) of the administered carbaryl dose was recovered as urinary 1-naphthol within 24 h post-dose; including the complete 48 h urine collection only increased the mean recovery to 23%. The total 1-naphthol levels quantified in 24 h urine collections ranged from 216 to 588 nmol/L (mean = 416; coefficient of variation (CV) = 39%), or with creatinine correction 21–84 µmol/mol creatinine (mean = 48.4; CV = 51%). Normalising these data for 70 kg body weight produced mean metabolite levels of 37.4 µmol 1-naphthol/mol creatinine (range 14.6–76.6; CV = 64%) (Figure 3). The 95% confidence interval for the 24 h total urinary 1-naphthol was 16–58 µmol/mol creatinine. Data for the individual volunteers is summarised in Table 2.

Levels of naphthol isomers quantified in post-shift urine samples obtained from workers exposed occupationally to naphthalene are presented in Table 3. The 1-naphthol levels ranged from below the limit of detection (<LoD) to 1027 µmol/mol creatinine with a median value of 4.2 µmol/mol creatinine (mean = 27.2). The 2-naphthol levels ranged from <LoD to 153 µmol/mol creatinine (median = 4.0, mean = 8.1). The highest levels of both naphthols (1027 and 153 µmol/mol creatinine for 1- and 2-naphthol, respectively) were detected in timber treatment workers, most likely from creosote.

## 4. Discussion

Urinary 1-naphthol is an established biomarker for assessing exposure to the insecticide carbaryl. However, to our knowledge, the data reported in this paper represent the first time that biomarker levels have been quantified in humans following administration of a known dose of carbaryl (at the ADI). Following an oral exposure to carbaryl, urine 1-naphthol levels rose rapidly, peaking between 2 and 6 h post-dose. The median excretion half-life was 3.6 h and elimination of 1-naphthol was almost complete within 24 h. Metabolite levels recovered in total urine collections for 24 h post-dose represented between 11% and 43% of the administered dose. We note that the two volunteers with the lowest recovery had the highest BMI. This could indicate that there is increased storage of carbaryl in body fat. However, there was no observable effect on the apparent excretion half-life in these individuals. Of further note is that the analytical method uses a glucuronidase hydrolysis step. While this will release free naphthol from glucurono-conjugates, other conjugates such as sulfates would not be accounted for.

The results presented here are useful for providing context for human biomonitoring data. Using the 24 h total urine collection data and correcting for a 70 kg individual, we would expect urinary 1-naphthol levels in the range of 16–58 µmol/mol creatinine following carbaryl exposure at the ADI. Concentrations in spot urine samples, more likely to be available for studies of occupational or environmental exposure, would be expected to be more variable and peak levels around 200 µmol 1-naphthol/mol creatinine might be expected, depending on the time between exposure and sampling.

A few studies have published levels of 1-naphthol in urine following occupational exposure to carbaryl. Bouchard et al. [9] reported urinary 1-naphthol levels up to about 200 µg/g creatinine (157 µmol/mol creatinine) in a small group of greenhouse workers. Petropoulou et al. [19] found 1-naphthol levels ranging from 1.4 to 20,444 ng/mL in urine from five farmers (approximately 0.8 to 11,784 µmol/mol creatinine assuming a litre of urine contains about 12 mmol creatinine [18]). Levels up to 9300 µg/g creatinine (7297 µmol/mol creatinine) were measured in a single farmer applicator [11]. 

Background levels of urine 1-naphthol in the UK population derived from the 95th percentile value were 12 µmol/mol creatinine [16]. Similar levels have been observed in the general population of Quebec [20] and in US and German populations [21,22]. Further, 1-naphthol is also a metabolite of naphthalene, which is a ubiquitous environmental pollutant. Consequently, it is difficult to isolate the relative contributions of carbaryl and naphthalene to the production of 1-naphthol in environmental exposure. However, the various background levels that have been reported are below levels that would be expected from dietary exposure to carbaryl at the ADI, even if we assume a worst case where all 1-naphthol metabolites arose from carbaryl exposure. Thus, we can conclude that exposure to carbaryl in the general population appears well controlled.

In addition to reporting the results from a human volunteer exposure study to carbaryl, some occupational exposure data for workers exposed to naphthalene has also been presented here. As described above, both carbaryl and naphthalene share 1-naphthol as a common metabolite, which in some circumstances can make identification of the source of exposure problematic. However, the ratio of 1- and 2-naphthol isomers in urine has been proposed as a means of distinguishing the exposure source, as the 2-isomer is only a metabolite of naphthalene [15]. The 1-/2-naphthol ratios ranging from 0.43 to 25.7 (5th and 95th percentiles) were reported by Meeker et al. [15] in environmentally exposed males. A similar range was found in this study for workers exposed to naphthalene (Table 3); of course, we have no way of estimating the carbaryl exposure of this group. However, limiting the data analysis to those samples where both naphthol isomers were greater than 4 µmol/mol creatinine (*n* = 127) did not significantly alter the distribution of the ratios. A qualitatively similar distribution of ratios was also found when limiting the analysis to samples containing 2-naphthol levels greater than the UK reference value (7.6 µmol/mol creatinine; [16]) (*n* = 87); indeed, about a third of these results had a 1-/2-naphthol ratio of greater than two (suggested by Meeker et al. (Approach 2) [15] as one way of identifying individuals with 1-naphthol primarily from carbaryl exposure). This indicates that there is considerable inter-individual variation in the relative production of naphthol isomers during naphthalene metabolism. Consequently, while the presence of 2-naphthol in a urine sample is confirmatory of exposure to naphthalene, we suggest that relatively greater levels of 1-naphthol do not necessarily indicate that carbaryl is the major contributor to exposure.

Within the occupational groups described here, chimney sweeps and aluminium smelters generally have low levels of exposure to naphthalene (>73% of samples within background range). Production and handling of coal tar products (including timber treatment) resulted in higher levels of exposure (>44% of samples exceed the background range) with some instances of significant exposure (17% of samples exceeded 200 µmol/mol creatinine for 1-naphthol).

## Figures and Tables

**Table 1:** Details of the study participants.

Code	Gender	Age	Height (m)	Weight (kg)	BMI
A	F	35	1.715	77	26.2
B	M	55	1.71	94	32.1
C	F	23	1.75	107	34.9
D	M	26	1.76	102	32.9
E	M	54	1.895	96	26.7

BMI: body mass index.

**Table 2:** Urinary excretion of 1-naphthol in five individuals administered an oral dose of carbaryl at the ADI. Data were calculated using 24 h total urine collection.

Code	Excretion Half-Life (h)	24 h Recovery (%)	Urinary 1-Naphthol (24 h Total)	
			nmol/L	µmol/mol Creatinine
A	2.5	43	572	84.3
B	3.3	27	397	48.1
C	2.7	11	588	57.9
D	4.7	11	308	21.3
E	4.8	17	216	30.3
**Mean**	**3.6**	**22**	**416**	**48.4**

**Table 3:** Levels of naphthol isomers (µmol/mol creatinine) found in 327 post-shift urine samples from 90 workers exposed to naphthalene. Isomer ratios are shown for (a) all samples with quantifiable levels of both isomers greater than the limit of detection (*n* = 233) and (b) samples with 2-naphthol levels greater than the environmental background reference value (*n* = 87).

Statistics	1-Naphthol	2-Naphthol	Ratio 1-/2-Naphthol ^a^	Ratio 1-/2-Naphthol ^b^
Min	<LoD	<LoD	0.07	0.07
25th percentile	<LoD	1.9	0.8	0.7
Median	4.2	4.0	1.4	1.3
75th percentile	13.4	8.6	3.0	2.8
Maximum	1027	153	33.6	14.2
Mean	27.2	8.1	-	-
Std deviation	105	14.8	-	-
*n*	*327*	*327*	*233*	*87*
